# Interactive Display of Images in Digital Exhibition Halls under Artificial Intelligence and Mixed Reality Technology

**DOI:** 10.1155/2022/3688797

**Published:** 2022-10-12

**Authors:** Xu Liu, Nan Zhang

**Affiliations:** ^1^Animation Art College, Jilin Animation Institute, Changchun, Jilin 130012, China; ^2^Department of Movie, Jilin Animation Institute, Changchun, Jilin 130012, China

## Abstract

The attractiveness of traditional exhibition halls to young people is gradually decreasing. Combining modern digital technology to improve the display effect of the exhibition hall can effectively enhance the effect of cultural publicity. This article introduces the technology of image interaction and mixed reality (MR) to improve the historical and cultural propaganda level of the Shaanxi exhibition hall. The advantages of MR technology in applying digital exhibition halls are theoretically expounded. A theoretical plan for Shaanxi history and culture-related display areas is designed using artificial intelligence combined with  MR  technology. In addition, the survey respondent's evaluation of the effect of the new exhibition hall is obtained using a questionnaire survey. The survey results show that 97% of people like the history and culture of Shaanxi but only 13% of the people say they know or know very well about the history and culture of Shaanxi. In addition, 60% of the tourists say they are satisfied with the cultural experience of Shaanxi, and only 27% of the tourists are very satisfied. Also, 96% of tourists are willing to experience Shaanxi's history and culture through digital exhibition halls, and 93% are willing to participate in cultural experience activities based on MR technology. The survey results prove that tourists are satisfied with the effect of the new exhibition hall. Tourists want to add a distinctive form of cultural experience to the exhibition hall. They are willing to accept digital exhibition halls incorporating MR technology and are very happy to participate in the exhibition method of image interaction. This shows that the use of image interactive display based on MR technology in the layout of the exhibition hall is recognized by people and has strong feasibility. This article has reference significance for the digital upgrade of the exhibition hall and the development of the cultural tourism industry.

## 1. Introduction

In the display method of exhibits, the traditional exhibition hall mainly displays through entities, with the text explanation and picture appreciation. This kind of display is dull and dreary, and its appeal to contemporary young tourists is gradually diminishing [1]. With the development of the times, the role of various exhibition halls is not limited to a single collection, research, and exhibition but highlights the role of education and cultural dissemination [2]. The digital exhibition hall display method that combines the traditional physical exhibition hall display method and mixed reality (MR) technology has become the development trend of the current exhibition hall with the integration and development of modern science and technology and display design; it has been used in various fields such as medical treatment, construction, education, and entertainment. Artificial intelligence (AI) is a branch of computer science. It is an interdisciplinary field of information that encompasses multiple disciplines. AI can develop theories, methods, techniques, and application systems for simulating, extending, and expanding human intelligence. It seeks to understand the nature of intelligence and produce a new type of intelligent machine that responds similarly to human intelligence. Since the birth of AI, theory and technology have become increasingly mature, and the application field has expanded. AI can simulate the information process of human consciousness and thinking.

International Business Machines Corporation cooperated with the Forbidden City as early as more than ten years ago. Tourists can deeply interactively learn about the Forbidden City using computer technology and an interactive experience system to create a virtual Forbidden City [3]. The Dunhuang Museum is planning to build an experiential tourist service center. Visitors can experience the Mogao Grottoes and feel the background culture of the Mogao Grottoes using advanced display forms [4]. The Louvre, the Metropolitan Museum, and the Palace of Versailles put their collections and paintings on the website. Tourists can visit these museums without leaving home using three-dimensional (3D) virtual technology [5]. Lin et al. researched and summarized MR image reconstruction based on deep learning (DL) in medical imaging [6]. They argued that DL-based algorithms could rival traditional reconstruction methods regarding image quality and computational efficiency. Zaharchuk and Davidzon found that AI could achieve rapid image acquisition and reconstruction and address the problems of AI-driven approaches in performing MR-based attenuation correction [7].

Currently, the number of digital exhibition halls using this technology is still small. This has reduced the development of the cultural tourism industry and the spread of various regional cultures to a certain extent. The Shaanxi exhibition hall still uses the traditional mode of operation and publicity. This article studies the interactive display method of images in the digital exhibition hall to improve the historical and cultural propaganda ability of the Shaanxi exhibition hall. A display area related to Shaanxi history and culture is designed using AI combined with MR technology. First, video interaction and MR technology are introduced in detail. Second, the necessity of applying MR technology in digital exhibition halls is expounded by revealing the advantages of digital exhibition halls compared to traditional exhibition halls. Third, the theoretical scheme of the interactive display of images in the digital exhibition hall is systematically planned. Finally, the feasibility of the interactive display method of digital exhibition hall images based on AI and MR technology is proved through a questionnaire survey. This article has reference value for the digital upgrade of relevant exhibition halls and the development of the cultural tourism industry.

## 2. Interactive Display of Images in Digital Exhibition Halls Based on AI and MR Technology

### 2.1. Image Interaction

“Interaction” means to communicate and interact. With the continuous development of current technology, its definition combines theories and methods of various disciplines such as immersion theory, computer application, and technological innovation and has become a common part of current life [8]. Image interaction is to adopt images as a presentation method. Various sensors can read the viewer's reaction, and real-time rendering technology is used to interact with the viewer [9].

**Figure 1:**
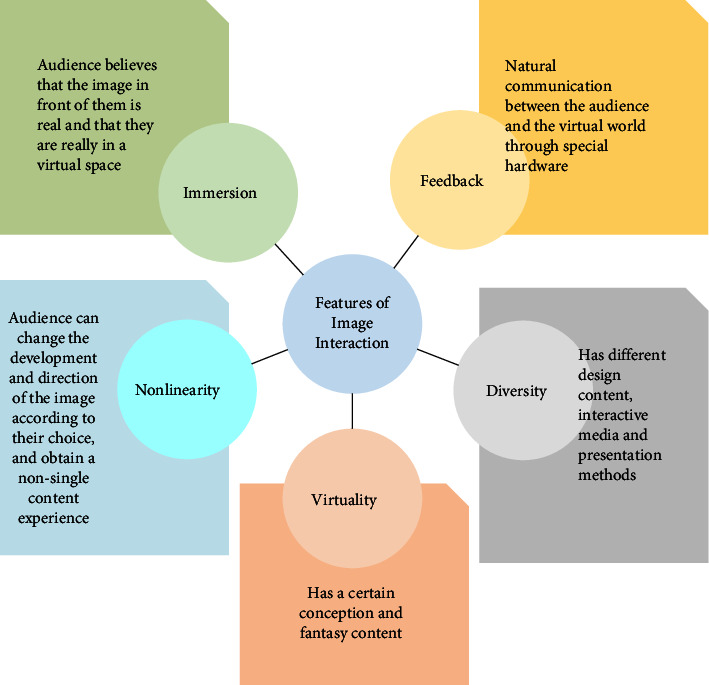
Features of image interaction.

Image interaction integrates media based on traditional images and has the features of traditional media and interaction [10]. The features of image interaction are shown in Figure 1.

From Figure 1, the features of image interaction are divided into five points, namely, immersion, feedback, diversity, virtuality, and nonlinearity [11, 12]. The specific performance of each feature is shown in Table 1.

### 2.2. MR Technology

VR technology uses a computer to simulate a virtual environment, so the experiencer is immersed in a 3D virtual space. The experiencer seems to be in the real world by mobilizing the experiencer's vision, hearing, and other senses [18]. The biggest feature of VR is that it gives the experiencer a strong sense of “immersion” and “presence.” Augmented reality (AR) is an extension of VR. It uses technologies such as computer vision to closely integrate virtual objects with the real world. The goal of AR is to enhance the experiencer's understanding of the real environment by combining virtual objects with the real environment [19]. MR is the combination of AR and VR, and the technology connects the user, the real world, and the virtual world in a way that enhances the user experience. The biggest feature of MR technology is that its processing and positioning of space are fine and accurate. Its calculations are performed in real-time. It combines virtual imagery with real space. It performs precise positioning according to its processing of images, thereby presenting a visual environment with both real and virtual images [20].  Table 2 reveals the features of MR technology.

### 2.3. The Necessity of Applying MR Technology in Digital Exhibition Halls

The traditional exhibition hall uses the display and placement transmission method to disseminate information through the physical exhibition, text explanation, picture appreciation, and model visit. The main visual design forms are exhibition boards, video broadcasts, and display props, which cannot break through the limitations of environmental space. The display method is rigid and boring, only staying in graphic design and lacking flexible design methods. The rich exhibition content cannot be presented [24]. In traditional exhibition halls, the uneven visual language and diverse information prevent visitors from making effective information choices. Too much emphasis is placed on the dominant position of the exhibits while ignoring the visitor's experience and feelings about the environment. In addition, the path that visitors choose to receive information is only static information presented by exhibits, pictures, and text. They do not form visual communication feedback with the display space. This single sensory communication interaction is mostly output in a one-dimensional pattern. There is no interaction between people and information, and visitors can only act as bystanders. Their aesthetic feeling is limited to static viewing and lacks a sense of communication experience [25].

According to the above shortcomings of traditional exhibition halls, the advantages of applying MR technology to digital exhibition halls are compared with them. This reflects the necessity of applying MR technology to digital exhibition halls. The specific comparison is demonstrated in Table 3.

### 2.4. Theoretical Scheme of Interactive Display of Images in Digital Exhibition Halls

**Figure 2:**
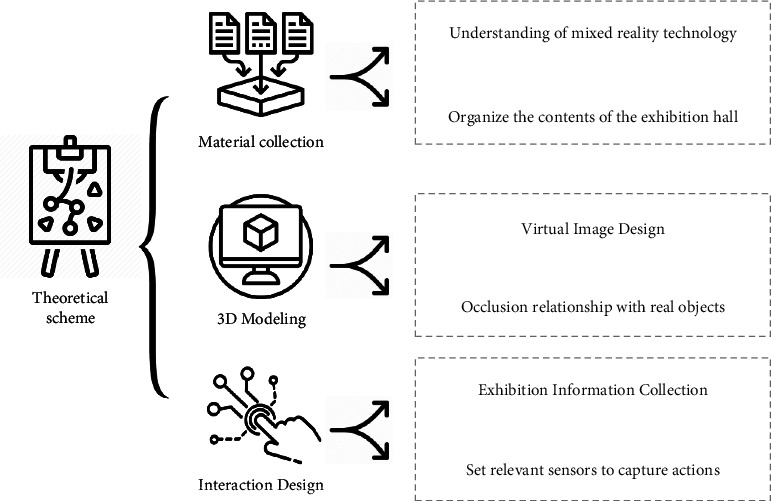
The theoretical scheme of interactive display of images in digital exhibition halls based on AI and MR technology.

The theoretical scheme of interactive images display in digital exhibition halls based on AI and MR technology is mainly divided into the material collection stage, 3D modeling stage, and interaction design stage [27], as shown in Figure 2.

The material collection stage includes the understanding of MR technology and the sorting out of the exhibition hall content. The understanding of MR technology includes understanding its definition and principle, the characteristics of MR technology, the development process, and the related hardware equipment and software support for realizing MR. It also delves into the performance methods and required technical conditions of MR technology. Sorting out the contents of the exhibition hall refers to the on-the-spot inspection of the exhibition hall and understanding the exhibition hall through photos, videos, and written records to form a complete system. According to the collected materials, they are classified and integrated and finally expressed on paper through sketches and flowcharts [28].

The 3D modeling stage deals with the occlusion relationship between virtual images and real exhibits in the real space through 3D modeling. This step requires rough depth estimation and precise contour processing by the image interaction system [29]. Therefore, when the overall occlusion environment is constructed, it is mainly divided into the following steps:

  Step 1: Model reconstruction of the target exhibit is performed. This step is conducive to accurately fitting the edge of the virtual image and the real object.
  Step 2: The position of the virtual model corresponding to the position of the real exhibits is marked and placed. The virtual coordinates and the physical coordinates of the real space are accurately aligned by the processor of MR technology. The position of the virtual image is used to simulate the occlusion objects in the real space to effectively occlude the virtual information.
  Step 3: The occlusion material is attached to the reconstructed digital model to make the digital model occluded.
  Step 4: The adjustment of the rendering parameters and the positional relationship between the front and rear of the virtual target makes the virtual object approach the occlusion effect of the real object in the display. Virtual imagery is used for precise localization and depth estimation of objects. Besides, scenes are built for MR spaces with occlusion relationships. In the mixed space, the real collections and virtual images have similar real edge occlusion and good interaction relationships.

**Figure 3:**
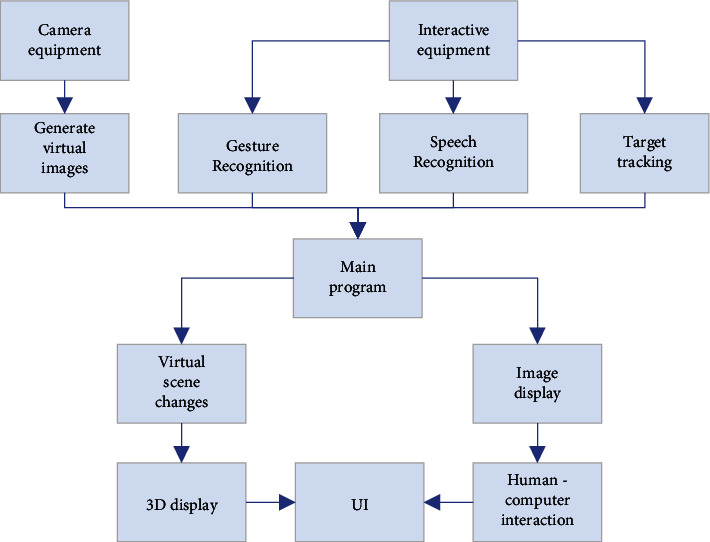
The overall framework of the system.

In the interaction design stage, the interactive process design of the digital exhibition hall image is mainly divided into two parts. In the first part, the characteristics of the exhibition hall and exhibits are collected through tracking and registration technology. The mixed virtual and real environment is effectively constructed and positioned. According to the system guidance, the target exhibits and the spatial relationship between the target exhibits and the entire environmental space are scanned, and data are loaded in advance, which helps the image interaction system input and read the coordinate information of the space. The image interaction system will construct a hybrid virtual space according to the obtained coordinate system. The occlusion relationship of the mixed space is constructed combined with vertex shaders in the mixed environment to improve the construction of the entire mixed environment [30].  Figure 3 displays the overall framework of the system.

In Figure 3, the data acquisition layer mainly collects image, speech, and human body coordinate data to provide basic data for subsequent panorama generation and display. The data processing layer is responsible for virtual image generation, gesture recognition, speech recognition, and target tracking. The user interface layer is responsible for displaying panoramic virtual images to realize human-computer interaction (HCI).

**Figure 4:**
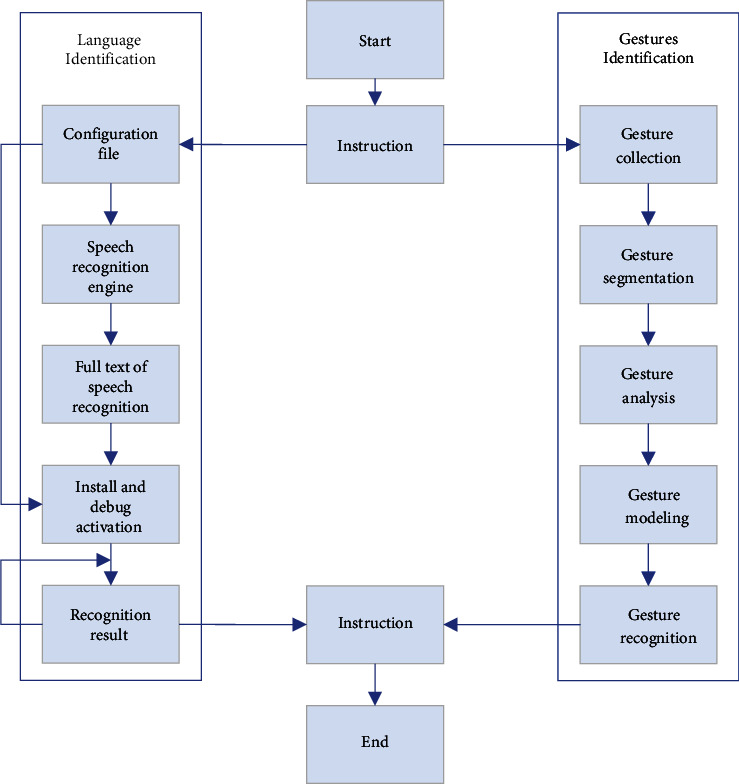
Instruction recognition program.

In the second part, the interaction mode of MR images is mainly based on user gestures, user gaze tracking, and visual locking. Gestures and sight lines are captured and acquired through the product's camera. A ray is emitted at the obtained coordinate position to determine the visual line of the user. The virtual information is encoded and set as a collidable material. The time is recorded when the simulated gaze line collides with the virtual information. The user's operation is made timely and effective. The specific operation steps of the instruction recognition program are shown in Figure 4.

This theoretical solution for the interactive display of images in digital exhibition halls applies to not only digital exhibition halls but also various museums. It can even be extended to all kinds of scenic spots and historical sites. The application of AI and MR technology in the digital exhibition hall is novel, intuitive, and highly interactive compared to the traditional exhibition hall display method. The scheme enhances the fun of the visit and deepens the understanding of the exhibits.

### 2.5. Questionnaire Survey

This article investigates the positioning and views of digital exhibition halls in people's minds by issuing questionnaires to ensure that the interactive display of images in digital exhibition halls based on AI and MR technology can be recognized and loved by public visitors. In this survey, the place where the questionnaires are distributed is located near the Shaanxi History Museum, mainly for the visitors who come to the museum. Visitors to the museum are randomly selected and complete a questionnaire after consent. Preinvestigation is conducted on five visitors, and the problems in the questionnaires and the inadequacy of the investigation are corrected. The test is conducted for two days. The specific content of the questionnaire is demonstrated in Table 4.

A total of 130 questionnaires are distributed, and 123 valid questionnaires are recovered, with an effective recovery rate of 94.62%.

The Cronbach's coefficient is used to test the reliability of the questionnaire results to examine the internal consistency of the scale.

(1) $$\begin{array}{c} \alpha =\frac{k\bar{r}}{1+\left(k-1\right)\bar{r}}\text{.} \end{array}$$

In equation (1), *k* is the number of evaluation indicators, $\bar{r}$ is the mean of the correlation coefficients of the *k* evaluation indicators, and *α* is between zero and one. The survey results are transformed according to the corresponding scores. The Cronbach's alpha of each index factor is obtained by inputting the results into the computer for SPSS processing. The scores of each index factor are screened to form the final index factor of the questionnaire. After calculation, the consistency of each Cronbach's alpha is within the acceptable range (0.8), indicating that the questionnaire results are highly reliable.

The content validity of the questionnaire is evaluated by the content validity ratio (CVR).

(2) $$\begin{array}{c} CVR=\frac{n-0.5N}{0.5N}\text{.} \end{array}$$

In equation (2), *n* is the number of questionnaires that the researchers believe can better reflect the measured items in the relevant questionnaire scoring results. *N* is the total number of returned questionnaires. The statistical result by SPSS software is 0.94, indicating that the overall questionnaire has high validity.

## 3. Survey Results and Analysis

### 3.1. Survey Results

**Figure 5:**
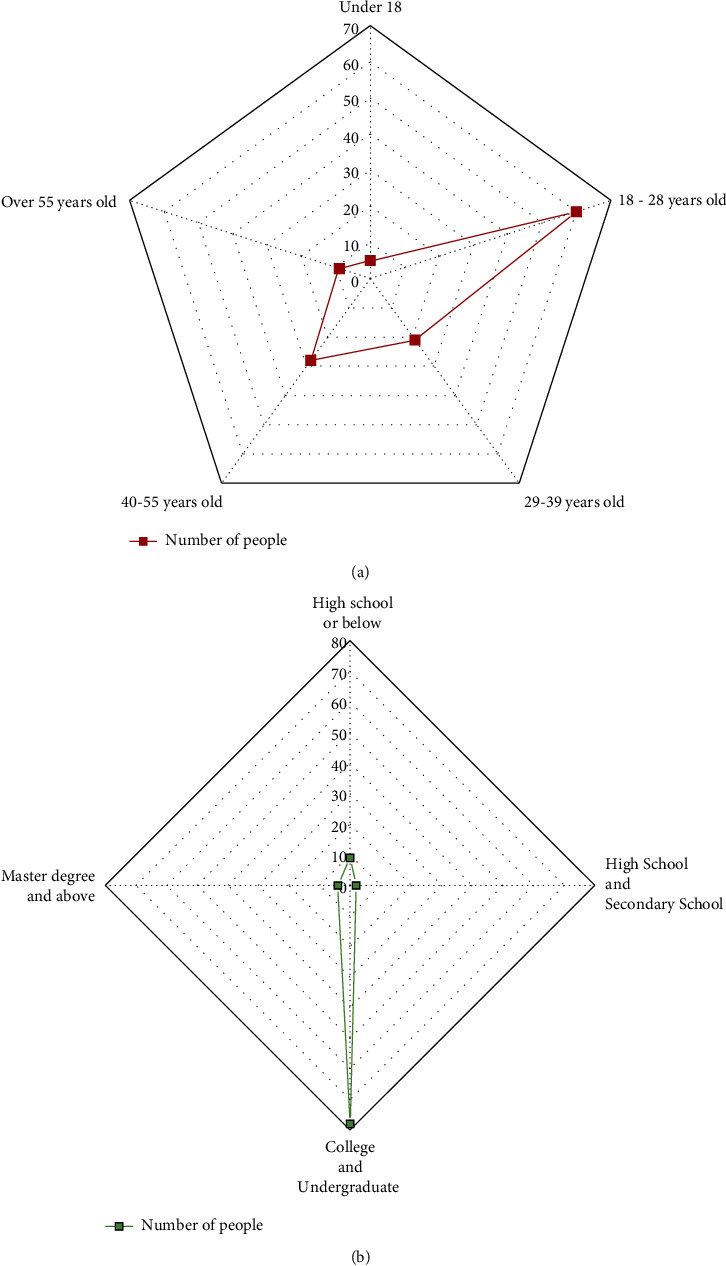
Distribution of age and education level of the surveyed population. (a) Age distribution of the surveyed population; (b) educational level distribution of the surveyed population.

In this questionnaire survey, the age and education level distribution of the surveyed population is shown in Figure 5.

From Figure 5, the age distribution of the respondents is 5 people under 18 years old, 60 people between 18 and 28 years old, 28 people between 29 and 39 years old, 21 people between 40 and 55 years old, and 9 people over 55 years old. They account for 3%, 50%, 23%, 17%, and 7% of the respondents, respectively. The education level of the surveyed population is 9 people from high school and below, 32 people from high school and technical secondary school, 78 people from junior college and undergraduate, and 4 people with a master's degree or above. They account for 7%, 27%, 63%, and 3% of the respondents, respectively.

**Figure 6:**
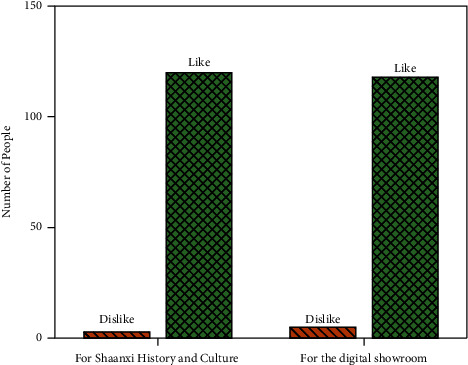
The degree of liking for Shaanxi's history and culture and the degree of liking for cultural experience among the surveyed people.

According to the questionnaire, the surveyed people's liking for Shaanxi's history and culture and their liking for cultural experience are shown in Figure 6.

From Figure 6, 97% of people like the history and culture of Shaanxi and only 3% dislike it. Besides, 96% are willing to experience Shaanxi history and culture through the digital exhibition hall, and only 4% are unwilling.

**Figure 7:**
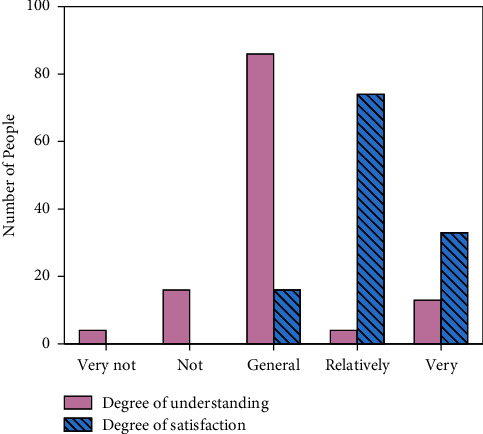
The surveyed population's understanding of Shaanxi's history and culture and their satisfaction with the local cultural experience.

Figure 7 reveals the degree of understanding of the people surveyed on Shaanxi's history and culture and their satisfaction with the local cultural experience.

From Figure 7, regarding the level of understanding of Shaanxi's history and culture, 10% say they know very well, 3% say they relatively understand, 70% say they are general, 13% say they do not understand, and 4% say they do not understand at all. According to the survey of satisfaction with Shaanxi cultural experience, 27% say they are very satisfied, 60% say they are relatively satisfied, 13% say they are general, and the number of dissatisfied people is zero.

**Figure 8:**
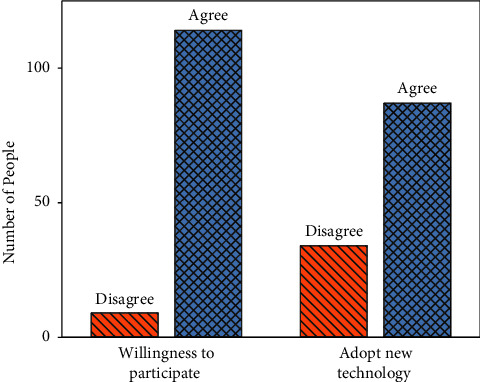
The willingness to participate in Shaanxi's historical and cultural experience and the percentage of respondents agreeing with the development direction of cultural tourism.

The proportion of respondents' willingness to participate in Shaanxi's historical and cultural experience and their approval of the development direction of cultural tourism is shown in Figure 8.

From Figure 8, 93% say they are willing to participate in cultural experience activities based on MR technology, and 7% say they are not. Among the expectations for the development of cultural tourism, 72% of the respondents express their agreement or strong agreement, and 28% say they are general or disagree.

### 3.2. Analysis of Results

According to the analysis of the above information, the tourists who come to Xi'an are very interested in the history and culture of Shaanxi. They are very willing to participate in the Shaanxi history and culture experience project, but the tourists who have arrived have a general understanding of the history and culture of Shaanxi. There are some obstacles to the spread of culture. Their satisfaction with the Shaanxi cultural experience in Xi'an is not high. Therefore, there is still room for improvement in the cultural experience project. For the development of the cultural tourism industry, tourists hope to have a new and characteristic form of cultural experience. People are willing to accept digital exhibition halls incorporating MR technology and are very happy to accept the exhibition method of image interaction. Bec et al. introduced the concept of second chance tourism and the role of innovative conservation methods such as virtual and MR [30]. This could reduce the pressure on inherently fragile tourist destinations, decrease the deterioration of tourist destinations, and create new tourism experiences. Bae et al. proposed a theoretical model based on brand equity theory in the context of MR experiences [31]. They surveyed 251 tourists visiting Seoul, South Korea's cultural and arts tourist attractions. The results showed that the characteristics of MR (interactivity and liveliness) not only affected the emotional aspects of the visitor experience (perceived immersion and enjoyment) but also positively impacted brand awareness, brand association, and brand loyalty. It is feasible to use the image interactive display method based on MR technology in the exhibition hall layout [32–37]. A conscious cultural communication behavior can be generated through the participation of tourists. This can not only facilitate the development of the cultural tourism industry in Xi'an but also play an important role in promoting the history and culture of Shaanxi.

### 3.3. Impact Analysis of AI and  MR  Technology

The use of MR technology in the interaction between exhibits and the audience has indeed improved the interactive experience under the premise of satisfying the display of important information and content of Shaanxi's history and culture [38]. In the space built by MR technology, the display of the situation is the most intuitive feeling when the viewer enters the MR display space. The sense of presence brought by the corresponding situation mainly drives the viewer's emotions [39–45]. The shaping and construction of the situation take the viewer's emotions and perspective as the main reference, mobilizing the viewer to generate many associations in the constructed virtual scene. The authenticity of the virtual scene fully improves the overall sense of substitution and the presence of the experiencers to mobilize their subjective initiative.

The HCI experience of the digital exhibition hall based on artificial intelligence enables the interactive operation between the experiencer and the technical equipment to jointly realize the intuitive information presentation of the mixed display effect, which is immersive. The experiencer can actively participate in the display of the digital exhibition hall to increase the enthusiasm and the fun of participation of the experiencer [46–51]. Therefore, these two technologies effectively improve the effect of the interactive display of images in digital exhibition halls in the sense of interaction and education.

## 4. Conclusion

This article studies the interactive display of images in digital exhibition halls based on AI and MR technology to promote the development of the cultural tourism industry across the country and strengthen the publicity and promotion of local cultures. Video interaction and MR technology are introduced in detail. Then, the necessity of applying MR technology in digital exhibition halls is expounded by revealing the advantages of digital exhibition halls compared to traditional exhibition halls. Furthermore, the theoretical scheme of the interactive display of images in the digital exhibition hall is systematically planned. Finally, a survey is conducted on 123 tourists who come to Shaanxi History Museum in Xi'an. The survey results show that 120 people like Shaanxi's history and culture, accounting for 97% of the total, but only 17 people say they know or know very well about Shaanxi's history and culture, accounting for only 13% of the total. Most tourists say they are satisfied with the cultural experience of Shaanxi. Only 33 tourists are very satisfied, accounting for 27%. Besides, 94% of tourists are willing to experience Shaanxi history and culture through digital exhibition halls, and 93% of tourists are willing to participate in cultural experience activities based on MR technology. The survey results imply that there is still room for improvement in the Shaanxi historical and cultural experience project in Xi'an, and tourists hope to have a new and characteristic form of cultural experience. People are willing to accept digital exhibition halls incorporating MR technology and are very happy to participate in the exhibition method of image interaction. Therefore, it is feasible to use the image interactive display method based on MR technology in the exhibition hall layout. This article only proposes a theoretical scheme for the interactive display of images in digital exhibition halls due to limited capabilities. Subsequently, these theoretical schemes will be applied in practice, and feasible practical schemes will be made. This article also has the following shortcomings and deficiencies. The new exhibition hall proposed here is only a theoretical concept, which needs to be implemented to become a specific new display method. In the future, it is necessary to combine design materials and decoration works to improve the feasibility of this theory. This article has certain reference significance for developing interactive displays of images in digital exhibition halls.

## Figures and Tables

**Table 1:** Feature performance of image interaction.

Feature	Specific performance
Immersion	Image interaction can bring the audience an immersive experience, and people enjoy sensory stimulation in the space expressed by images. This breaks through the traditional single visual sensory experience. Image interaction achieves full integration and immersion with the support of projection technology, sensing technology, image acquisition, sound technology, and other technologies and enters people's psychological space to gain a strong sense of participation and satisfaction [13].

Feedback	The participants get visual, auditory, and informational feedback for the first time to communicate and interact when they contact the works. In the virtual reality (VR) experience, people participate in the virtual environment through body movements, gesture changes, eye gaze, and voice control. Besides, interactions between scenes generate. People feel the grip, weight, and movement of real objects in the virtual environment, and the virtual images and participants can get interactive feedback in the experience [14].

Diversity	On one hand, the diversity of interactive images is reflected in the fusion of various media. The media produced by the development of science and technology and the interaction between media can form a whole. On the other hand, there are various forms of interaction. Image interaction can produce the interaction between the artist and the work, the audience and the work, the work and the environment, and the work and the work. Image interaction is an active process that presents various interactive modes [15].

Virtuality	Image interaction simulates the real environment through digital technology. The audience wears VR glasses to participate in the VR scene. People see digital virtual space through visual experience. The real action in the virtual environment can change development in the virtual environment and affect people's emotions to achieve the realism of virtual experiences. Virtuality is characterized by a sense of interlacing between virtual and real space-time, which escapes the constraints of time and space and increases the audience's perceptual experience [16].

Nonlinearity	The interactive experience is innovative in the traditional video linear narrative method. The spontaneous dissemination of the audience is based on the strong feeling of the heart. Image interaction is different from the single clue narrative of traditional works of art. It can extend various aspects of story clues and generate thinking through a single point. Everyone will have different spiritual perceptions and artistic results when they participate. The cognition of nontraditional art theory is a nonlinear occurrence to generate the possibility of multiple narratives [17].

**Table 2:** Features of MR technology.

Feature	Specific performance
Reality	MR can combine virtual and reality to create an immersive feeling. The experiencer feels a new environment that is half real and half virtual to produce a good sensory experience [21].
Conceptuality	In a realistic new environment, users use their imagination to construct an environment that does not exist objectively. MR technology has a very large room for development. MR will continue to enrich the interactive display technology of images in digital exhibition halls with the continuous development and improvement of technologies such as computers and VR [22].
Interactivity	MR technology combines VR with traditional physical displays, building a bridge between virtual objects and the real world. A connection between users and physical objects is established. Users can move freely between reality and VR, change the traditional static display state, and break the one-way nature of information transmission to realize real-time interaction and communication between people and objects [23].

**Table 3:** Comparison of traditional exhibition halls and digital exhibition halls.

Disadvantages of traditional exhibition halls	Advantages of digital exhibition halls	The necessity of MR technology in the digital exhibition hall
Single display method	The innovative form of popular science	At present, most of the exhibition halls in China still have many problems, such as the same and single display content and form. These problems seriously restrict the sustainable development and construction of Chinese museums. Many exhibition halls have adopted technologies such as multimedia audio, touch screen, video storytelling, interactive games, and hand-held interactive devices. However, the application of VR technology, which is very popular in recent years, is less in exhibition halls. In particular, the application of MR technology is almost nonexistent, so MR technology is applied to the digital exhibition hall. This move not only enriches the connotation of the exhibits but also innovates the display form of the exhibits [26].

Passive information reception	The audience's understanding and mastery of knowledge	MR uses features such as virtual-real integration, situational simulation, special sound effects, and real-time interaction to inject new vitality into the exhibition hall. The transmission of sound can enhance the appeal of the displayed content and reproduce the historical situation. The real objects are presented in front of the audience in all directions, and the interaction with the audience can be realized. The audience can zoom in and rotate the images of the exhibits according to their needs to understand the history of the exhibits or their connotations. This form is no longer limited to a single and distant display. The audience is subtly influenced by this form of presentation. The audience deepens the knowledge and understanding of the exhibits [26].

Insufficient communication and interaction	Audience's high interest	The MR display form uses new science and technology to let the audience enter the virtual situation based on the original cabinet display. The audience interacts with exhibits in many audio effects. The exhibits present not only the exquisite appearance but also the accumulation of cultural knowledge. The new display form of MR is helpful to highlight the details of the exhibits and enrich the content of the exhibits. The realization of MR technology makes up for the shortcomings of traditional display forms in terms of time and space transformation, emotional penetration, and multisensory participation. This can stimulate the audience's interest in visiting and enhance the educational effect of the museum [26].

**Table 4:** Visitor questionnaire.

Question number	Questions and options									
1	Gender	□Male		□Female						

2	Age									
	□Under 18	□18–28		□29–39			□40–55		□Over 55	

3	Education level									
	□High school and below	□High school and technical secondary school		□Junior college and undergraduate			□ Master's degree or above			

4	Identity									
	□Staff of government agencies/institutions						□Company boss/employee			
	□Scientific research/education/medical workers			□Student			□Soldier		□Retiree	
	□Others (please specify)									

5	Travel method									
	□Travel organized by travel agencies			□Free travel						

6	Do you like Shaanxi culture?			□Yes			□No			

7	Do you feel the historical and cultural experience of Shaanxi in Xi'an?						□Yes		□No	

8	Understanding of Shaanxi history and culture									
	□Not at all	□Do not understand		□Generally			□Understand		□Understand well	

9	Evaluation of Xi'an tourism and cultural experience									
	□Very satisfied	□Satisfied		□Generally			□Dissatisfied		□Very dissatisfied	

10	Are you willing to participate in the Shaanxi historical and cultural experience?						□Yes		□No	

11	Favorite form of cultural experience									
	□Cultural exhibition hall	□Cultural experience hall		□Humanistic performance			□Interactive experience		□Cultural attractions	

12	Reasons that affect your experience of Shaanxi history and culture									
	□Like natural scenic spots			□Poor publicity						
	□Do not pay attention			□Very concerned about and love the history and culture of Shaanxi						
	□Cultural experience is traditional									

13	Are you satisfied with the form and content of the current cultural experience?						□Yes		□No	

14	Would you like to participate in cultural experience activities using MR technology?									
	□Yes						□No			

15	Expectations for the development of the cultural tourism industry									
			Agree very much		Agree	Generally		Disagree		Very disagree

Hope there are many new forms										

Desire to develop a unique cultural experience										

Hope the new technology can be applied to cultural tourism										

## Data Availability

The raw data supporting the conclusions of this article will be made available by the authors, without undue reservation.

## References

[1] Khan M. A., Israr S., S Almogren A., Din I. U., Almogren A., Rodrigues J. J. P. C. (2020). Using augmented reality and deep learning to enhance taxila museum experience. *Journal of Real-Time Image Processing*.

[2] Krzywinska T., Phillips T., Parker A., Scott M. J. (2020). From immersion’s bleeding edge to the augmented telegrapher: a method for creating mixed reality games for museum and heritage contexts. *Journal on Computing and Cultural Heritage*.

[3] Margetis G., Apostolakis K. C., Ntoa S., Papagiannakis G., Stephanidis C. (2020). X-reality museums: unifying the virtual and real world towards realistic virtual museums. *Applied Sciences*.

[4] Lee H., Jung T. H., tom Dieck M., Chung N. (2020). Experiencing immersive virtual reality in museums. *Information & Management*.

[5] Vosinakis S., Nikolakopoulou V., Stavrakis M., Fragkedis L., Chatzigrigoriou P., Koutsabasis P. (2020). Co-design of a playful mixed reality installation: an interactive crane in the museum of marble crafts. *Heritage*.

[6] Lin D. J., Johnson P. M., Knoll F., Lui Y. W. (2021). Artificial intelligence for MR image reconstruction: an overview for clinicians. *Journal of Magnetic Resonance Imaging*.

[7] Zaharchuk G., Davidzon G. (2021). Artificial intelligence for optimization and interpretation of PET/CT and PET/MR images. *Seminars in Nuclear Medicine*.

[8] Elkadi H., Al-Maiyah S., Fielder K., Kenawy I., Martinson D. B. (2021). The regulations and reality of indoor environmental standards for objects and visitors in museums. *Renewable and Sustainable Energy Reviews*.

[9] Sugiura A., Kitama T., Toyoura M., Mao X. (2019). The use of augmented reality technology in medical specimen museum tours. *Anatomical Sciences Education*.

[10] Aguayo C., Eames C., Cochrane T. (2020). A framework for mixed reality free-choice, self-determined learning. *Research in Learning Technology*.

[11] Ercan F. (2020). An examination on the use of immersive reality technologies in the travel and tourism industry. *Business & Management Studies: International Journal*.

[12] Emeafor O., Onyemechalu S. (2021). Objectivity in museums: the nigerian civil war according to the national war museum, umuahia. *The International Journal of the Inclusive Museum*.

[13] Hammady R., Ma M., Strathern C., Mohamad M. (2020). Design and development of a spatial mixed reality touring guide to the egyptian museum. *Multimedia Tools and Applications*.

[14] Bertrand S., Vassiliadi M., Zikas P., Geronikolakis E., Papagiannakis G. (2021). From readership to usership: communicating heritage digitally through presence, embodiment and aesthetic experience. *Frontiers in Communication*.

[15] Suroto P. Z., Dewantara M. H., Wiradarmo A. A. (2020). The application of technology in museums. *International Journal of Applied Sciences in Tourism and Events*.

[16] Shehade M., Stylianou-Lambert T. (2020). Virtual reality in museums: exploring the experiences of museum professionals. *Applied Sciences*.

[17] Torres-Ruiz M., Mata F., Zagal R., Guzmán G., Quintero R., Moreno-Ibarra M. (2020). A recommender system to generate museum itineraries applying augmented reality and social-sensor mining techniques. *Virtual Reality*.

[18] Reading A., Bjork J., Hanlon J., Jakeman N. (2021). The labour of place: memory and extended reality (xr) in migration museums. *Memory Studies*.

[19] Camps-Ortueta I., Deltell-Escolar L., Blasco-López M. F. (2021). New technology in museums: ar and VR video games are coming. *Communications Society*.

[20] Cardoso P. J. S., Rodrigues J. M. F., Pereira J. (2020). Cultural heritage visits supported on visitors’ preferences and mobile devices. *Universal Access in the Information Society*.

[21] Vokshi A., Shehu E., Dervishi S. (2021). Military archaeology and contemporary reality in Albania. *City, Territory and Architecture*.

[22] Condell J., Mcshane N., Avlarez J., Miller A. (2021). Virtual community heritage – an immersive approach to community heritage. *The Journal of Media Innovations*.

[23] Fairchild A. J., Campion S. P., García A. S., Wolff R., Fernando T., Roberts D. J. (2017). A mixed reality telepresence system for collaborative space operation. *IEEE Transactions on Circuits and Systems for Video Technology*.

[24] Chalhoub J., Ayer S. K. (2018). Using mixed reality for electrical construction design communication. *Automation in Construction*.

[25] Strzys M. P., Kapp S., Thees M. (2017). Augmenting the thermal flux experiment: a mixed reality approach with the hololens. *The Physics Teacher*.

[26] White M., Petridis P., Liarokapis F., Plecinckx D. (2007). Multimodal mixed reality interfaces for visualizing digital heritage. *International Journal of Architectural Computing*.

[27] Birt J., Stromberga Z., Cowling M., Moro C. (2018). Mobile mixed reality for experiential learning and simulation in medical and health sciences education. *Information*.

[28] Osipov I. V. (2017). Cubios transreality puzzle as a mixed reality object. *International Journal of Virtual and Augmented Reality*.

[29] Frajhof L., Borges J., Hoffmann E., Lopes J., Haddad R. (2018). Virtual reality, mixed reality and augmented reality in surgical planning for video or robotically assisted thoracoscopic anatomic resections for treatment of lung cancer. *The Journal of Visualized Surgery*.

[30] Wu X., Rong L., Jie Y., Song X., Cao Y., Yang S. (2018). Mixed reality technology launches in orthopedic surgery for comprehensive preoperative management of complicated cervical fractures. *Surgical Innovation*.

[31] Bec A., Moyle B., Schaffer V., Timms K. (2021). Virtual reality and mixed reality for second chance tourism. *Tourism Management*.

[32] Bae S., Jung T. H., Moorhouse N., Suh M., Kwon O. (2020). The influence of mixed reality on satisfaction and brand loyalty in cultural heritage attractions: a brand equity perspective. *Sustainability*.

[33] Liu R., Wang X., Lu H. (2021). SCCGAN: style and characters inpainting based on CGAN. *Mobile Networks and Applications*.

[34] Cao B., Zhao J., Lv Z., Yang P. (2021). Diversified personalized recommendation optimization based on mobile data. *IEEE Transactions on Intelligent Transportation Systems*.

[35] Zhou W., Yu L., Zhou Y., Qiu W., Wu M. W., Luo T. (2018). Local and global feature learning for blind quality evaluation of screen content and natural scene images. *IEEE Transactions on Image Processing*.

[36] Li D., Ge S. S., Lee T. H. (2022). Simultaneous arrival to origin convergence: sliding-mode control through the norm-normalized sign function. *IEEE Transactions on Automatic Control*.

[37] Zhu B., Zhong Q., Chen Y. (2022). A novel reconstruction method for temperature distribution measurement based on ultrasonic tomography. *IEEE Transactions on Ultrasonics, Ferroelectrics, and Frequency Control*.

[38] Wu X., Zheng W., Chen X., Zhao Y., Yu T., Mu D. (2021). Improving high-impact bug report prediction with combination of interactive machine learning and active learning. *Information and Software Technology*.

[39] Cai T., Yu D., Liu H., Gao F. (2022). Computational analysis of variational inequalities using mean extra-gradient approach. *Mathematics*.

[40] Wang Z., Dai L., Yao J. (2021). Improvement of Alcaligenes sp. TB performance by Fe-Pd/multi-walled carbon nanotubes: enriched denitrification pathways and accelerated electron transport. *Bioresource Technology*.

[41] Lin Y., Song H., Ke F., Yan W., Liu Z., Cai F. (2022). Optimal caching scheme in D2D networks with multiple robot helpers. *Computer Communications*.

[42] Zhang X., Wang Y., Yang M., Geng G. (2021). Toward concurrent video multicast orchestration for caching-assisted mobile networks. *IEEE Transactions on Vehicular Technology*.

[43] Zhou W., Wang H., Wan Z. (2022). Ore image classification based on improved CNN. *Computers & Electrical Engineering*.

[44] Yan J., Jiao H., Pu W., Shi C., Dai J., Liu H. (2022). Radar sensor network resource allocation for fused target tracking: a brief review. *Information Fusion*.

[45] Ban Y., Liu M., Wu P. (2022). Depth estimation method for monocular camera defocus images in microscopic scenes. *Electronics*.

[46] Wang Q., Zhou G., Song R., Xie Y., Luo M., Yue T. (2022). Continuous space ant colony algorithm for automatic selection of orthophoto mosaic seamline network. *ISPRS Journal of Photogrammetry and Remote Sensing*.

[47] Zheng W., Liu X., Yin L. (2021). Research on image classification method based on improved multi-scale relational network. *PeerJ Computer Science*.

[48] Ma Z., Zheng W., Chen X., Yin L. (2021). Joint embedding VQA model based on dynamic word vector. *PeerJ Computer Science*.

[49] Zheng W., Yin L., Chen X., Ma Z., Liu S., Yang B. (2021). Knowledge base graph embedding module design for Visual question answering model. *Pattern Recognition*.

[50] Qi M., Cui S., Chang X. (2022). Multi-region nonuniform brightness correction algorithm based on L-channel gamma transform. *Security and Communication Networks*.

[51] Jiang S., Zhou J., Qiu S. (2022). Digital agriculture and urbanization: mechanism and empirical research. *Technological Forecasting and Social Change*.

